# The Effect of Four-Month Training on Biochemical Variables in Amateur Cross-Country Skiers

**DOI:** 10.3390/jcm13206026

**Published:** 2024-10-10

**Authors:** Natalia Grzebisz-Zatońska

**Affiliations:** Department of Human Biology, Faculty of Physical Education, Józef Piłsudski University of Physical Education in Warsaw, 00-968 Warsaw, Poland; n.grzebisz@gmail.com

**Keywords:** training, cross-country skiing amateur, biochemistry, morphology

## Abstract

**Background/Objectives**: Research on the effects of training on the health of amateur cross-country skiers remains limited, particularly on biochemical parameters. Therefore, this study aimed to assess such changes in response to four months of training for amateur cross-country skiers. **Methods**: Blood samples were collected after spring training in May (active rest) and in September at the end of the preparatory period. The Wilcoxon signed-rank test was used for comparison of both measurements (initial and final). **Results**: Training in a group of amateur cross-country skiers exerts a statistically significant influence on the decrease in leukocyte count (thousand/µL) (*p* = 0.045) and the decrease in mean corpuscular hemoglobin concentration (MCHC) (g/dL) (*p* = 0.021). The other parameters did not show statistically significant changes. **Conclusions**: The change in MCHC and WBC can be attributed to adaptation to prolonged exercise, with a simultaneous increase in VO_2_ max. It should not be identified with pathology. The decrease in MCHC was likely attributable to changes in hydration status and a slight reduction in hemoglobin concentration. Similarly, it is recommended that other biochemical parameters be within the reference ranges.

## 1. Introduction

Activity requiring physical effort is a key element of a healthy lifestyle that can guarantee improvement and maintenance of health. However, the relationship between them are still being assessed and debated [1,2]. While the effects of long-duration efforts of various disciplines on health have been assessed previously [3,4,5,6,7], there is still much to be discovered about the effects of long-distance cross-country skiing (XCS) in middle-aged amateurs. While there are studies in the professional group [8,9,10], there is a clear need for more analyses in the largest group of competitors—amateurs. As XCS is ranked as one of the most exhausting sports [11], it is of the utmost importance to monitor the effects of exertion on the body to protect health and ensure the optimal development of exercise capacity. There has been a notable enhancement in the performance capabilities of amateur competitors in sports events. This is evidenced by the narrowing of the time differential between them and the top-performing professionals (as recorded in the Ski Classics series). This can be attributed to advancements in their running technique, enhanced capacity to leverage the capabilities of their equipment, and improved effort capabilities. The Worldloppet series records an approximate total of 65,000 participants in its competitive events throughout a single winter season [12]. Additionally, the number of professional teams is increasing, thus improving the overall level of participants, the quality of equipment or training, and its preparation [13,14,15]. This also applies to those engaged in amateur skiing who receive the support of trainers, physiotherapists, doctors, physiologists, and biochemists. In this group, it is crucial to monitor their health. In contrast to professionals, the training effects in amateurs are not sufficiently monitored and evaluated. Despite the growing popularity of long-distance XCS, research into its impact on health remains limited [16,17]. This is also the case concerning the hematological profile of this group. While there are several papers on the impact of activity on this cohort, none have assessed the impact on fundamental biochemical parameters after the preparatory period [12,18,19,20].

It is important to determine the impact of training on the hematological profiles of amateur XCS, as current knowledge on this topic is limited. This could be valuable for further optimizing training strategies and competitions.

The aim of this study was to evaluate the impact of four months of training on biochemical parameters in this group.

## 2. Materials and Methods

On 22 January 2019, the examination received approval from the Warsaw University of Life Sciences (SGGW) Bioethics Committee at the Faculty of Human Nutrition and Consumption (Nr. 38p/2018) and was conducted according to the guidelines outlined in the Good Clinical Practice and the Helsinki Declaration. Before the commencement of the tests, all participants were asked to submit written consent. The first test was conducted after spring training in May, the second in September at the end of the preparatory period.

### 2.1. Participants

The study involved 16 male, amateur XCS. They lived in big cities and mostly had sedentary jobs.

Inclusion criteria: consent to participate, valid medical consent, and finishing at least three races last season with a minimum length of 40 km. It was a prerequisite for participants to be in a state of complete health, free from any disease. The participants in the study were amateurs who had volunteered to take part in the research project and to follow a training plan. The loads were completed by the individual energy zones identified during the preliminary time trial test (cardio-pulmonary exercise test). Additionally, only male participants were included in the study. Given the potential hormonal fluctuations during the menopausal transition in women, as well as the observed differences in VO_2_ max, body weight, body composition, and hemoglobin levels, it was deemed necessary to limit the study to a single sex to enhance the homogeneity and matching of the group.

Exclusion criteria: no consent to participate in the study, the occurrence of any disease, or no medical green light. In addition to the aforementioned criteria, females, along with those suffering from illness, having sustained injuries, lacking agreement with the training plan, and having declined to participate in the study, were also excluded.

### 2.2. Anthropometric Measurements

Right before the test was carried out, Tanita MC-980 MA Plus Body Composition Analyzer (Arlington Heights, IL, USA) was used to measure the body weight, as well as body mass index (BMI) and fat mass (% and kg). Tests were performed in Sportslab Sports Diagnostics Center, Warsaw, Poland.

### 2.3. Biochemical Variables

Venous blood samples were taken on an empty stomach in the morning (7 to 10 am). Cobas 8000 (Basel, Switzerland) and spectrophotometric methods were used to determine the variables: iron, alanine aminotransferase (ALT), aspartate aminotransferase (AST), creatinine, gamma-glutamyl transpeptidase (GGTP), urea, uric acid, magnesium, total calcium, amylase, alkaline phosphatase (ALP), and glucose and total bilirubin. Alifax (Polverara, Italy) and the automatic methodology allowed us to measure of erythrocyte sedimentation rate (ESR). Potassium, and lipid profile indicators (high-density lipoprotein-cholesterol (HDL), low-density lipoprotein-cholesterol (LDL), triglycerides, and total cholesterol) were measured with the spectrophotometric method. The immunoturbidimetric method determined C-reactive protein (CRP), and thyroid stimulating hormone (TSH), testosterone and cortisol were measured with the electrochemiluminescence immunoassay (ECLIA) method.

### 2.4. VO_2_ Max Test

To assess the VO_2_ max, a time trial test (cardio-pulmonary exercise test) was employed. It was advised that prolonged and intense exercise should be avoided for 72 h before the test. A time trial test was conducted on a treadmill HP Cosmos CPET (Nussdorf-Traunstein, Germany) apparatus and Cosmed Quark/k4B2 (Rome, Italy). During the test, heart rate, oxygen uptake, exhaled carbon dioxide, and other parameters of the cardiorespiratory system were measured. Before the commencement of the study, the weight and body composition of the amateur participants were surveyed to obtain information regarding their body mass, which was then used to determine their VO_2_ max. The participant commenced the trial at a speed of 6 km/h with the treadmill at 0% inclination. Subsequently, every 3 min the speed increased by 1 km/h and the inclination by 1% until the subject reached a state of exhaustion or refused to continue. Tests allowed the determination of VO_2_ max. It was achieved when VO_2_ increase uptake stopped or slowly significantly, despite the increase in running speed. Lactate levels were also determined via the collection of fingertip blood samples before, during, and following each episode of the test. This information was later used to determine individual energy zones and lactate thresholds (D-max method). The frequency of heart contractions at rest and during exercise was recorded using the Garmin ANT+ (Olathe, KS, USA) heart rate monitor. The VO_2_ max results of the test are presented in this study. All measurements were carried out at the Sportslab Sports Diagnostics Center in Warsaw, Poland.

### 2.5. Training Loads

From June to September, the participants completed a training schedule defined by a professional XCS coach. It was based on the periodization of sports training and the requirements of the discipline. The training plan mainly consisted of endurance and resistance training and the workouts were planned for five days per week—two circuit training to develop endurance and strength), and to of endurance training. One session was planned for complementary training. The endurance session lasted 1.5–2.5 h and resistance 1–1.5 h, depending on the training period. In total, 60% of the targeted training was carried out on roller skis, 25% on ski trainers, and 15% was devoted to ski imitation.

The initial time trial test indicated the presence of individual intensity zones for each amateur.

The effort was performed in six zones.

Zone I (active regeneration)—active rest zone. Effort in this zone supports an active form of recovery after intensive efforts (swimming, yoga, stretching, core stability training).

Zone II (low intensity) is a low-intensity zone. Effort in it increases endurance capacity in conditions of moderate fatigue. That is, e.g., cycling (a long session with low intensity), or ski trainer workout (aimed at strengthening core muscles and arms). These two zones depend on the metabolism of free fatty acids to produce energy.

AT—medium intensity zone. Training with this intensity aims to increase the ability to perform aerobic (aerobic) efforts. It increases the burning of fat tissue, e.g., running, resistance circuit workouts, ski imitations, or roller skiing.

The submaximal high-intensity zone is characterized by a sustained level of exertion that is above the individual’s lactate threshold but below the point of maximum oxygen uptake. Such exertion encourages the adaptation to the anaerobic efforts that are typical of competitive conditions. The substrates that facilitate energy acquisition within this metabolic zone are phosphocreatine, glucose from the blood, glycogen, and ATP (adenosine triphosphate).

The term “Max” is used to describe the maximum intensity zone. It promotes adaptation to short-term efforts of maximum intensity up to 60 s, typical for acceleration. Its basis is in phosphocreatine and ATP substrates.

**Figure 1:**
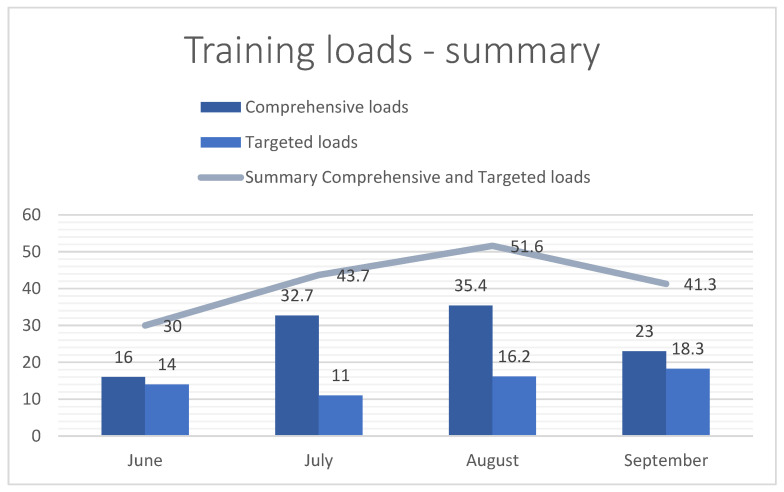
Summary of training loads in hours.

**Figure 2:**
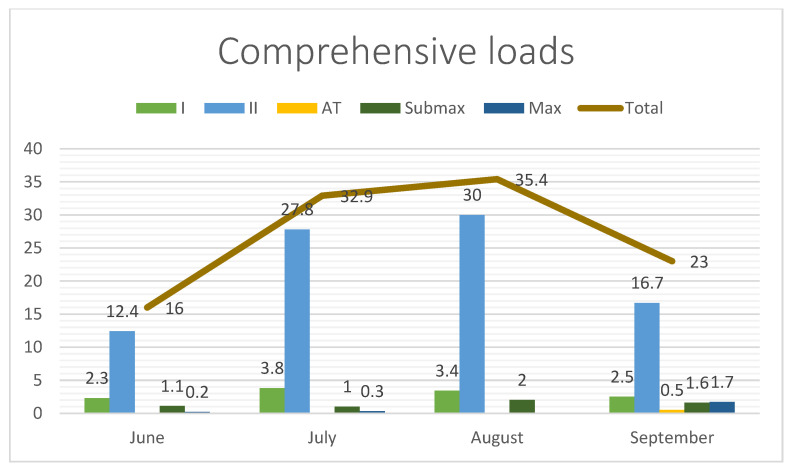
Summary of comprehensive loads in hours and intensity.

**Figure 3:**
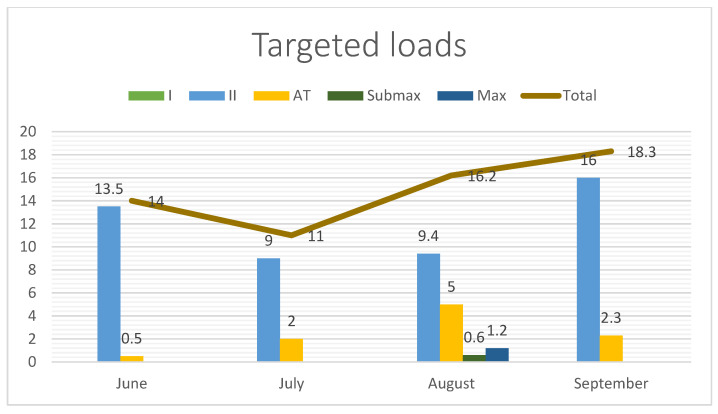
Targeted loads in hours per month and intensity.

Figure 1, Figure 2 and Figure 3 show the total training loads (in hours) as well as the time spent on comprehensive effort (swimming, running, cycling, general development exercises) and the targeted training (roller skiing, ski trainer, and imitation). The figures also show the intensity and training loads in the test period. The intensity was divided into five zones: Zone I, Zone II (aerobic), Zone AT (anaerobic threshold) (mixed), Zone Submax (anaerobic submaximal), and Zone Max (anaerobic maximal).

### 2.6. Statistics

Statistica 13.1 software (StatSoft, Tulsa, OK, USA) for MS Windows 10 was used to make calculations. The data was analyzed using basic descriptive statistics. The value of 0.05 was assumed to represent the significance. The Wilcoxon signed-rank test was used to compare the results from the initial and final tests. The significance level was assumed to be * *p* < 0.05.

## 3. Results

The initial test resulted in the following somatic parameters: age 37.5 ± 6.6 years, weight 79.8 ± 6.3 kg, height 182.2 ± 6.0 cm, body mass index (BMI) 23.7 ± 1.3 kg/m^2^, body fat mass (kg) 12.1 ± 2.6, % body fat mass 15.3 ± 2.6.

The somatotype parameters in the second test, after the training period, were as follows: age 37.9 ± 6.8 years, weight 78.4 ± 6.0 kg, height 182.2 ± 6.0 cm, body mass index (BMI) 23.6 ± 1.2 kg/m^2^, body fat mass (kg) 11.4 ± 2.7, and % body fat mass 14.6 ± 3.0. There were statistically significant changes in fat mass (kg and %, *p* = 0.023 and *p* = 0.038, respectively).

During the first time trial test, the VO_2_ max was 49.0 ± 4.5 mL/kg/min, while after the second test, this value was 51.1 ± 4.6 mL/kg/min. This was a statistically significant change (*p* = 0.008).

Table 1 compares the biochemical results of the initial and final tests. The results show statistically significant changes in leukocyte count (WBC) (thousand/µL) and MCHC (g/dL). None of the other parameters showed statistically significant changes. MPV (increase *p* = 0.075) and neutrophil count (decrease *p* = 0.079) were observed to be close to statistical significance. Statistical significance equal to 1 was noted for red blood cell distribution width-standard deviation (RDW-SD), basophils (thousand/µL) and neutrophils (%), non-HDL cholesterol (mg/dL), and testosterone (ng/dL). An equally small change (*p* > 0.9) was recorded for eosinophils (%), basophils (%), aspartate transaminase (AST) (U/L), potassium (mmol/L), iron (µg/dL), and C-reactive protein (CRP) (mg/dL) (Table 1).

## 4. Discussion

The most important observations from the study are statistically significant changes in WBC (thousand/μL) and MCHC (g/dL). No changes (statistically significant) were noted in the remaining parameters. MPV (increase) and neutrophil count (decrease) were observed to be close to statistical significance. It is also noteworthy that there was an increase in VO_2_ max, which occurred concurrently with a lack of statistically significant changes in the remaining measured parameters (except WBC and MCHC). All biochemical indicators were within the reference ranges for the population, which is a particularly important finding. The results of these studies indicate that training for cross-country skiing amateurs during the main preparation period does not result in significant changes. Furthermore, individuals who engage in increased physical activity do not exhibit discernible differences in their biochemical blood profiles compared to the general population. However, monitoring the response and adaptation to the physical effort is essential.

This is also confirmed by a statistically significant increase in VO_2_ max. It is also interesting to note that there was no statistically significant change in CRP (*p* = 0.925). It is generally known that endurance training, unlike high-intensity intermittent exercise, does not contribute to the secretion of this pro-inflammatory factor [21]. Its significant increase is observed in major homeostasis disorders, in inflammatory states, and in response to poor adaptation to exercise. Although beyond the scope of this manuscript, it is worth noting that fat mass may be a factor that indicates the correct response of the body to exercise and its pro-health effect. In these studies, there was a statistically significant decrease in measurements in kilograms and percentages (*p* = 0.023 and *p* = 0.038, respectively). The direction of these changes was correct and within the reference ranges for the population and physically active people [8,18]. This result confirms that the loads were chosen correctly and had a positive impact on the amateur’s health.

In this study, significant changes were observed in WBC and MCHC, which may indicate adaptation to exercise loads. However, further analysis is required to determine whether this is an adaptive process or pathology.

### 4.1. Leukocytes

It is established that biochemical parameters, which are also related to the immune system, are sensitive to exercise and physical training. However, this depends on the intensity and volume of the training. Notable changes are observed, particularly when the individual’s capacity to maintain homeostasis is exceeded, such as during overtraining, prolonged and intense exercise, or hypoxia [22,23]. This is associated with the endocrine and nervous system response, as well as the subsequent inflammatory process [21]. However, post-recovery leukocytosis is transient, and after 24-h rest, it returns to baseline level [24]. The impact of training (chronic exercise) on immune parameters, particularly in non-professional athletes, is still not well understood.

In adult healthy populations, the WBC is typically within the range of 4000 to 10,000 per microliter (µL) of blood. In our study, this parameter was close to the lower limit, with a value of 5.4 (0.9) in the first study and 5.0 (0.8) in the second. Resting WBC may be lower in people practicing selected endurance sports than in the general population. A similar response was recorded for neutrophils, with a statistically insignificant decrease (*p* = 0.079) and a range within the range for the normal population. Such a difference was observed in aerobic sports compared to team or technical sports [25]. Furthermore, the same authors proposed that this phenomenon may be an adaptive response to prolonged aerobic exercise, rather than an underlying pathology.

In the context of these studies, while a notable reduction in WBC in response to the training regimen was observed, with values approaching the lower threshold, this does not automatically signify an underlying pathological state. Instead, these findings may reflect an adaptation to exercise. Similar observations have also been documented in professional athletes and other social groups [25,26,27,28].

As the reduced WBC count was within the normal range, it can be concluded that the four-month training used in this study does not negatively affect immune function in amateur XCS.

It is important to highlight the accessibility of research findings. The impact of various stimuli on the immune system in the general population is well documented. However, the challenge remains to assess this variability in the group of professional athletes, despite an increasing number of available publications. However, they are still lacking in the amateur group. These findings may help to fill this gap. A systematic review by Ciro Alexandre Mercês Gonçalves et al. [17] has shown that both acute and chronic interventions are able to modify most of the immunological markers. However, this response can be affected by gender, physical capacity, environment, type of exercise, and intensity.

A reduction in white blood cell (WBC) count was not identified as a factor associated with the illness in this study. However, it is recommended that consideration be given to the possibility of supporting the immune system, given that other factors that occur in the autumn (after the period studied) may have an impact. It is widely acknowledged that excessive and prolonged effort can precipitate immune depression. Furthermore, an unbalanced diet, inadequate hydration, stress, poor quality of sleep, and contact with sick people increase the risk of infection. These variables should be particularly monitored during the autumn in cross-country skiing amateurs. In the event of exceeding reference ranges, these athletes may develop diseases that negatively affect their exertion capabilities.

A signal that immune capacity is impaired is when reference ranges are exceeded. This may increase susceptibility to infections (including upper respiratory tract infections, URTIs) in athletes and negatively affect their exercise capacity. This may be a consequence of excessive training loads or may be due to additional variables, as indicated earlier. In practice, it is crucial to prevent significant and prolonged decreases or increases above the WBC reference, which has disrupted homeostasis and compromised health, thereby compromising adequate exercise capacity.

They emphasize that the number of relationships between the immune system and physical training means that the immunology of exercise is still poorly understood. Understanding these relationships would be valuable for proper training plans and health protection. More research is therefore needed in this area.

### 4.2. Mean Corpuscular Hemoglobin Concentration

The reference interval for the MCHC is 32–36 g/dL. Results out of this range usually indicate anemia or iron deficiency. However, this parameter itself is not an indication of this disease. It is important to assess other indications in the RBC profile.

MCHC is not a typical diagnostic test but the ratio of hemoglobin mass to hematocrit. Optimal values ensure efficient oxygen binding [29,30]. The results of these tests, although showing a statistically significant decrease, were within the reference norm. This was also the case for WBC. The MCHC values obtained were higher than in other studies, but only slightly, and do not cause clinical problems in amateurs. In the study by Lippi et al. [31], it was 33.2 (pg/L) in non-active people, 32.5 in amateur cyclists, and 33.3 in professional cyclists. Other results showed that the hematological blood parameters of physically active and inactive people did not differ significantly, but were within the reference limits [32]. Their results are therefore more similar to those of professional athletes than inactive people.

In these studies, hematocrit also increased, and hemoglobin decreased slightly (changes not statistically significant). MCHC is known to decrease with a decrease in red blood cell count or low hemoglobin, which was not observed. This can also be seen in iron, vitamin B9, and B12 deficiency. The most likely cause of the statistically significant change in MCHC in these studies is related to the enlarged plasma volume that occurs with dehydration. This is a possible cause of this change. However, it is important that this change was within the reference range and does not indicate pathology. It would be worthwhile to include an additional assessment of hydration in the future.

The overall variability in Hgb and Hct, and therefore MCHC, may also be due to differences in training level, exercise dose, and duration, plasma volume levels, training frequency and intensity, gender, diet, and age. It was confirmed by Ciekot-Sołtysiak M et al. [33]. The authors noted that MCH and MCHC values are higher in triathletes than in sprinters after the transition and preparation training period, but not after the competition period. They may be influenced by prolonged aerobic exercise, as in this study. In addition, Ciekot-Sołtysiak M et al. [33] showed that hematological parameters in athletes change statistically significantly over year-round training, but are within reference ranges. These conclusions are consistent with these studies. Variations were only observed between the different training phases, indicating the need to monitor blood parameters every three months. In the future, it would be worthwhile to carry out research with a group of amateurs throughout the annual training cycle (12 months).

In practice, biochemical monitoring should also focus on the parameters of the red blood cell system and MCHC components. A reduction in the concentration of hemoglobin, red blood cells, iron, vitamin B9, and vitamin B12 beyond the reference range may have a significant impact on MCHC. This will result in a reduction in the potential for physical exercise.

Also noteworthy in these studies is the increase in Mean platelet volume (MPV). A similar trend has been observed in previous studies [34,35]. It also predicts performance in middle-distance running [36] and is derived from moderate exercise [37].

Although this requires further research, likely, that improved fitness is also associated with increased MPV, which was the case in our study. This is also worth investigating in future projects, as it has not been confirmed in all other studies [38].

### 4.3. Limitations and Future Research

A limitation of the study is the relatively small sample size. It would be beneficial to include amateurs from other countries in future interventions, as this may yield more valuable results. It should be noted that the research did not include women. To gain a more accurate and reliable understanding of the impact of exercise on health status, further research is required. This may be of particular interest in the context of middle-aged menopausal women. It would also be valuable to include a control group without an exercise intervention, with a different workload, or people with hypercholesterolemia. The results of the study in a group of professional athletes evaluate the changes in the annual training cycle. Extending the study period and increasing the number of measurements would also be valuable to gain a better understanding of how amateur cross-country skiers’ exercise affects their body condition. Further research is therefore needed to determine how physical training affects blood biochemical parameters in different groups of physically active people. Such data are still lacking. The increasing popularity of long-term physical activity may require reference ranges for its safe effects on the body.

## 5. Conclusions

This study aimed to assess the impact of changes in hematological markers in cross-country skiers during the preparatory phase of the macrocycle. Training in a group of amateur cross-country skiers exerts a statistically significant influence on the decrease in leukocyte count (thousand/µL) (*p* = 0.045) and the decrease in mean corpuscular hemoglobin concentration (MCHC) (g/dL) (*p* = 0.021). The other parameters did not show statistically significant changes. The change in MCHC and WBC can be attributed to adaptation to prolonged exercise, particularly because of the increase in VO_2_ max. However, this does not indicate the presence of pathology. A reduction in white blood cell (WBC) count was not associated with the manifestation of immunological dysfunction. The alteration in MCHC was likely attributable to changes in hydration status and a slight reduction in hemoglobin concentration. Therefore, the red blood cell system should be monitored, with particular attention paid to hemoglobin, red blood cells, iron, vitamins B9, and B12 that affect them, and hydration. In response to amateur training, an increase in VO_2_ max should be noted, and a simultaneous decrease in WBC and MCHC may indicate adaptation to exercise. It is similarly advised that other biochemical parameters be within the reference ranges.

A larger group of participants, including control groups, women, and changes in the annual training cycle, could be analyzed in future research.

## Figures and Tables

**Table 1:** Hematological results of the initial and final tests.

Parameter	Result 1 Mean (SD)	Result 2 Mean (SD)	*p*-Value
Leukocytes (WBC) (thousand/µL)	5.4 (0.9)	5.0 (0.8)	0.045
Erythrocytes (M/µL)	5.1 (0.3)	5.0 (0.3)	0.666
Hemoglobin (g/dL)	15.2 (0.5)	15.0 (0.9)	0.414
Hematocrit %	43.8 (1.6)	44.0 (2.7)	0.780
Mean corpuscular value (MCV) (fL)	88.0 (3.5)	89.1 (3.8)	0.103
Mean corpuscular hemoglobin (MCH) (pg)	30.5 (1.0)	30.3 (1.0)	0.209
Mean corpuscular hemoglobin concentration (MCHC) (g/dL)	34.7 (0.9)	34.1 (0.7)	0.021
Platelets (thousand/µL)	211.1 (34.9)	207.6 (43.7)	0.414
Red blood cell distribution width-standard deviation (RDW-SD) (fL)	42.1 (3.9)	41.9 (3.3)	1.000
Red blood cell distribution width-coefficient of variation (RDW-CV) %	13.0 (0.8)	13.0 (0.9)	0.724
Platelet distribution width (PDW) (fL)	13.6 (2.2)	13.8 (2.6)	0.432
Mean platelet volume (MPV) (fL)	10.7 (1.1)	10.8 (1.1)	0.075
Platelet–large cell ratio (P-LCR) %	32.4 (8.8)	32.3 (8.4)	0.889
Procalcitonin (PCT) %	0.3 (NA)	0.2 (NA)	NA
Neutrophils (thousand/µL)	2.7 (0.6)	2.4 (0.5)	0.079
Lymphocytes (thousand/µL)	2.0 (0.6)	1.9 (0.6)	0.209
Monocytes (thousand/µL)	0.4 (0.2)	0.4 (0.1)	0.859
Eosinophils (thousand/µL)	0.3 (0.2)	0.3 (0.4)	0.306
Basophils (thousand/µL)	0.0 (0.0)	0.0 (0.0)	1.000
Neutrophils %	49.8 (8.2)	49.2 (7.7)	1.000
Lymphocytes %	38.2 (7.7)	37.7 (6.9)	0.551
Eosinophils %	4.8 (3.0)	4.4 (1.7)	0.919
Basophils %	0.6 (0.4)	0.6 (0.4)	0.906
ESR (mm/h)	4.8 (3.7)	5.0 (3.8)	0.615
Urea (mg/dL)	33.7 (6.6)	35.8 (9.4)	0.232
Uric acid (mg/dL)	5.6 (1.5)	5.6 (1.1)	0.470
Glucose (mg/dL)	86.8 (17.0)	86.5 (11.4)	0.660
Total cholesterol (mg/dL)	179.3 (33.7)	182.5 (39.4)	0.286
HDL (mg/dL)	57.9 (12.5)	62.5 (14.5)	0.551
Non-HDL (mg/dL)	120.2 (38.6)	109.2 (42.7)	1.000
LDL (mg/dL)	106.2 (32.2)	110.0 (36.0)	0.233
Triglycerides (mg/dL)	80.0 (35.2)	76.4 (37.6)	0.572
AST (U/L)	29.1 (23.6)	23.2 (5.7)	0.916
ALT (U/L)	21.7 (7.5)	21.0 (7.0)	0.463
ALP (U/L)	59.8 (10.5)	57.2 (10.5)	0.106
GGTP (U/L)	19.0 (10.4)	20.2 (12.5)	0.674
Serum amylase (U/L)	64.2 (22.0)	63.3 (19.4)	0.484
Sodium (mmol/L)	141.3 (2.2)	140.6 (1.8)	0.326
Potassium (mmol/L)	4.5 (0.4)	4.5 (0.5)	0.944
Total calcium (mmol/L)	2.4 (0.1)	2.9 (1.9)	0.442
Magnesium (mmol/L)	0.8 (0.1)	1.0 (0.4)	0.432
Iron (µg/dL)	110.9 (52.1)	113.5 (27.2)	0.950
CRP (mg/dL)	0.7 (1.0)	0.6 (0.4)	0.925
TSH (µIU/mL)	1.8 (0.7)	1.0 (0.8)	0.414
Testosterone (ng/dL)	599.1 (216.8)	619.8 (207)	1.000
Cortisol (µg/dL) 7–10 AM	14.7 (4.2)	15.0 (3.4)	0.834
Total bilirubin (mg/dL)	1.8 (2.3)	0.9 (0.5)	0.167
Granulocytes (thousand/µL)	0.7 (0.2)	0.7 (0.4)	0.414
Monocytes (%)	7.5 (2.9)	8.8 (1.9)	0.224

## Data Availability

Data are available on request due to privacy and ethical restrictions.
